# Effectiveness of the GPT-4o Model in Interpreting Electrocardiogram Images for Cardiac Diagnostics: Diagnostic Accuracy Study

**DOI:** 10.2196/74426

**Published:** 2025-08-22

**Authors:** Haya Engelstein, Roni Ramon-Gonen, Avi Sabbag, Eyal Klang, Karin Sudri, Michal Cohen-Shelly, Israel Barbash

**Affiliations:** 1 Sheba Medical Center, Tel Hashomer, Ramat Gan, Israel; 2 The Graduate School of Business Administration, Information Systems Program, Bar-Ilan University, Max and Anna Webb St, Ramat Gan, 5290002, Israel, 972 3-531-8910; 3 Davidai Arrhythmia Center, Sheba Medical Center, Tel Hashomer, Ramat Gan, Israel; 4 Faculty of Medicine, Tel Aviv University, Tel Aviv, Israel; 5 Division of Data-Driven and Digital Medicine (D3M), Icahn School of Medicine at Mount Sinai, New York, NY, USA; 6 The Charles Bronfman Institute of Personalized Medicine, Icahn School of Medicine at Mount Sinai, New York, NY, USA; 7 Sheba ARC, Sagol Big Data and AI Hub, Sheba Medical Center, Tel Hashomer, Ramat Gan, Israel; 8 Interventional Cardiology Unit, Leviev Heart Center, Sheba Medical Center, Tel Hashomer, Ramat Gan, Israel

**Keywords:** artificial intelligence, cardiology, decision support systems, electrocardiogram, large language models, LLMs

## Abstract

**Background:**

Recent progress has demonstrated the potential of deep learning models in analyzing electrocardiogram (ECG) pathologies. However, this method is intricate, expensive to develop, and designed for specific purposes. Large language models show promise in medical image interpretation, and yet their effectiveness in ECG analysis remains understudied. Generative Pretrained Transformer 4 Omni (GPT-4o), a multimodal artificial intelligence model, capable of processing images and text without task-specific training, may offer an accessible alternative.

**Objective:**

This study aimed to evaluate GPT-4o’s effectiveness in interpreting 12-lead ECGs, assessing classification accuracy, and exploring methods to enhance its performance.

**Methods:**

A total of 6 common ECG diagnoses were evaluated: normal ECG, ST-segment elevation myocardial infarction, atrial fibrillation, right bundle branch block, left bundle branch block, and paced rhythm, with 30 normal ECGs and 10 of each abnormal pattern, totaling 80 cases. Deidentified ECGs were analyzed using OpenAI’s GPT-4o. Our study used both zero-shot and few-shot learning methodologies to investigate three main scenarios: (1) ECG image recognition, (2) binary classification of normal versus abnormal ECGs, and (3) multiclass classification into 6 categories.

**Results:**

The model excelled in recognizing ECG images, achieving an accuracy of 100%. In the classification of normal or abnormal ECG cases, the few-shot learning approach improved GPT-4o’s accuracy by 30% from the baseline, reaching 83% (95% CI 81.8%-84.6%). However, multiclass classification for a specific pathology remained limited, achieving only 41% accuracy.

**Conclusions:**

GPT-4o effectively differentiates normal from abnormal ECGs, suggesting its potential as an accessible artificial intelligence–assisted triage tool. Although limited in diagnosing specific cardiac conditions, GPT-4o’s capability to interpret ECG images without specialized training highlights its potential for preliminary ECG interpretation in clinical and remote settings.

## Introduction

Artificial intelligence (AI) in the realm of medicine, including cardiology, has been consistently evolving. A significant recent AI milestone was achieved when a model, specifically ChatGPT by OpenAI, successfully passed the European Exam in Core Cardiology [1]. However, this evaluation focused solely on text-based multiple-choice questions, excluding those with audio or visual elements. While this accomplishment is impressive, cardiology heavily relies on image interpretation and visual data for patient assessment [2].

Deep learning (DL), which uses neural networks for image-related tasks [3], has already demonstrated its significant impact in medical image analysis, including cardiology [4,5]. Moreover, it has been proven effective in predicting clinically significant abnormalities in electrocardiograms (ECGs), such as potassium levels and adverse reactions to medications, while also extracting valuable insights beyond human capabilities, such as estimating sex, age, and identifying specific cardiac conditions [6-10]. For example, Prifti et al [7] trained convolutional neural networks (CNNs) on short ECG recordings to accurately detect early signs of drug-induced cardiac effects and inherited rhythm disorders. In a separate study, Attia et al [9] demonstrated that deep CNNs could estimate a person’s age and sex solely from the heart’s electrical signals, tasks that even experienced cardiologists cannot perform reliably, highlighting AI’s ability to uncover hidden insights from routine medical data. However, while DL has shown great promise, developing a DL model requires substantial efforts, including the collection of large, labeled datasets and extensive training for the specific task [11,12].

Large language models (LLMs), such as Generative Pretrained Transformer, specialize in processing human language using artificial neural networks [13]. The newly introduced multimodal LLM, GPT-4 Omni (GPT-4o) by OpenAI, advances this even further by seamlessly combining text and image data, presenting substantial potential benefits in the medical domain [14-18].

In emergency rooms, efficient patient triaging based on ECG findings is crucial. An AI model capable of distinguishing between normal and abnormal ECGs, even without offering a specific diagnosis, holds significant promise for improving patient care. The concept of “ECG triage” has the potential to transform how patients are prioritized for cardiology consultations.

This study aims to evaluate the ability of general purpose LLMs to interpret ECG images using zero-shot and few-shot learning strategies across a range of diagnostic tasks, including ECG recognition, binary classification (normal vs abnormal), and multiclass pathology classification. Our goal is to determine whether GPT-4o can perform these tasks with sufficient accuracy to support its potential role in clinical ECG triage and diagnosis.

## Methods

### Image Collection and Cohort Selection

**Figure 1.**
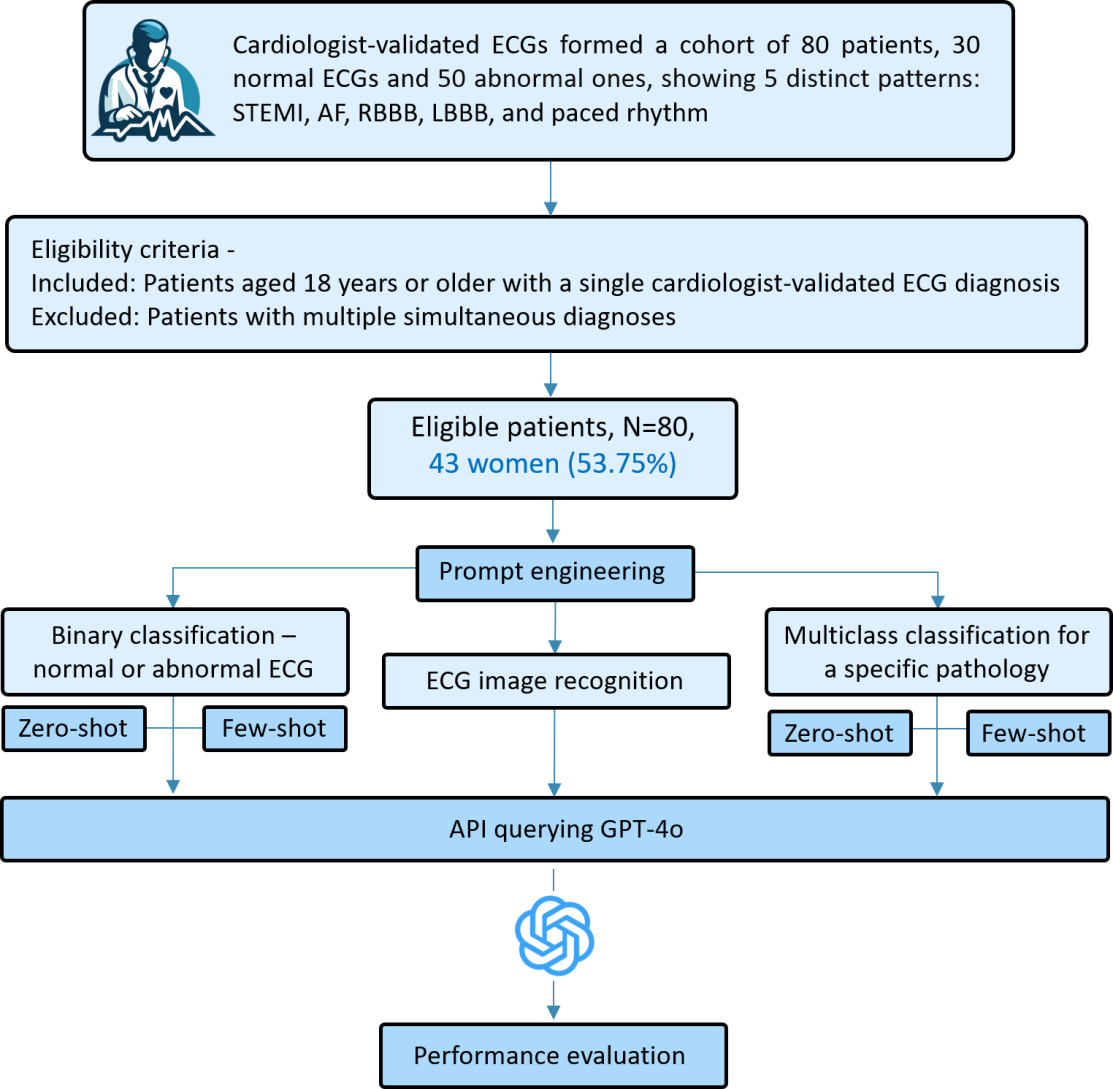
Research methodology overview illustrating the research methodology and portraying the high-level design of the method. AF: atrial fibrillation; API: application programming interface; ECG: electrocardiogram; GPT: generative pretrained transformer; LBBB: left bundle branch block; RBBB: right bundle branch block; STEMI: ST-segment elevation myocardial infarction.

The study design is depicted in Figure 1.

The study included patients aged 18 years or older who underwent a high-quality ECG recording using the MUSE (GE HealthCare Technologies) system at our institute from August 2010 to February 2024.

A cohort of 80 arbitrarily chosen 12-lead ECG strips was assembled, covering 6 distinct electrocardiographic presentations. This included 30 records of normal ECG strips and an additional 50 ECG strips representing 5 distinct, common diagnoses (10 ECG strips of each different diagnosis): ST-segment elevation myocardial infarction (STEMI), atrial fibrillation (AF), right bundle branch block (RBBB), left bundle branch block (LBBB), and paced rhythm. These pathologies were chosen for their diverse representation of cardiac conditions, each with unique electrocardiographic features [19]. All ECG charts were anonymized, removing age and gender identifiers.

### Data Validation

Each case underwent thorough validation via electronic medical record review, with ECG findings meticulously interpreted by a board-certified cardiologist. Only those patients with a singular diagnosis for each condition were included to ensure study validity; those with multiple diagnoses or low-quality images were excluded.

### GPT-4o Prompt Engineering and Study Design

GPT-4o is a state-of-the-art multimodal model proficient in analyzing both image and text inputs. We used the OpenAI API to test whether it can interpret ECG images and classify them accurately into distinct categories. We tested three main scenarios: (1) Can GPT-4o recognize an ECG image? (2) Can GPT-4o classify an ECG image as normal or abnormal? (3) Can GPT-4o classify an ECG image into 1 of the 6 specific diagnoses: normal ECG, AF, STEMI, LBBB, RBBB, and paced rhythm?

#### Learning Techniques

In scenarios 2 and 3, we evaluated 2 learning approaches—zero-shot and few-shot [20]. The zero-shot approach involved providing the model with only a textual instruction describing the classification task, without any previous examples. In contrast, the few-shot approach included a limited number of ECG images, each labeled with its diagnosis, to serve as training data [21,22]. These examples were intended to guide the model in recognizing diagnostic visual patterns and applying them when analyzing new ECGs. To ensure unbiased testing, the evaluation excluded images used for training. For example, if 6 images were given as examples, 54 images were evaluated. This design optimizes training efficiency.

#### Prompt Formats

In some scenarios, we repeated the same task using three different prompt formats to assess how varying levels of complexity and detail affect model performance. The formats were (1) a basic prompt stating only the classification task, (2) a prompt that included the task along with brief descriptions of each class, and (3) a detailed prompt that combined the task with explicit textual guidance, instructing the model on specific visual features to consider when analyzing the ECG images.

#### Experimental Scenarios’ Processes

The following section outlines the procedures and objectives of each experimental scenario designed to evaluate GPT-4o’s ability to interpret ECG images. Table 1 shows the different experiments conducted across the 3 tested scenarios, and Multimedia Appendix 1 provides the exact prompts used in each experiment.

**Table 1.** Experiments description^a^.

Experiment	Scenario	Technique	Task	Total, N	Few-shot training sample	Testing sample
1.1	1	Zero-shot	Recognize ECG^b^	60	0	60
1.2	1	Zero-shot	Classify ECG or not ECG	60	0	60
2.1	2	Zero-shot	Classify normal or abnormal. No textual guidance.	60	0	60
2.2	2	Zero-shot	Classify normal or abnormal. Minimal textual guidance.	60	0	60
2.3	2	Zero-shot	Classify normal or abnormal. Textual guidance was provided.	60	0	60
4.2	2	Few-shot	Classify normal or abnormal—learn 6 examples. No textual guidance.	60	6	54
4.3	2	Few-shot	Classify normal or abnormal—learn 6 examples along with added textual guidance.	60	6	54
4.4	2	Few-shot	Classify normal or abnormal—learn 10 examples along with added textual guidance.	60	10	50
3.1	3	Zero-shot	Classify into 6 classes (normal and 5 pathologies). No textual guidance.	60	0	60
3.2	3	Zero-shot	Classify into 6 classes (normal and 5 pathologies). Textual guidance was provided.	60	0	60
5.1	3	Few-shot	Classify into 6 classes (normal and 5 pathologies). Examples were provided.	60	6	54
5.2	3	Few-shot	Classify into 6 classes (normal and 5 pathologies). Examples were provided along with added textual guidance.	60	6	54

a The table summarizes the experimental design, including the scenario, prompting technique, classification task, total number of images used, and the number of examples provided in few-shot learning settings.

b ECG: electrocardiogram.

### Scenario 1: ECG Image Identification

This scenario aimed to evaluate the GPT-4o model’s ability to recognize ECG images. The dataset included 60 ECG images, each assessed individually by the model. Two experiments were conducted: the first (experiment 1.1) was a simple test to determine whether GPT-4o could recognize that the image presented was an ECG, using the prompt “What is this image? Output one line for the label.” The second experiment (experiment 1.2) explicitly asked the model to classify the image as either “ECG” or “not ECG.”

### Scenario 2: Distinguishing ECG Images as Normal or Abnormal

This scenario aimed to evaluate the GPT-4o model’s ability to distinguish between normal and abnormal ECG images. The dataset included 30 normal and 30 abnormal ECGs (6 images from each of the 5 abnormalities). Using the zero-shot approach, ECGs were presented without previous examples or guidance. For few-shot learning, 3 experiments were conducted (4.2, 4.3, and 4.4). Two experiments used a single composite image made up of 6 examples (3 normal and 3 abnormal), with and without textual guidance. In the third experiment, 2 composite images with textual guidance were used, together containing 10 examples (5 normal and 5 abnormal). Each file contained a mix of normal and abnormal examples (Table 1 and Multimedia Appendix 1).

### Scenario 3: Multiclass Classification for a Specific Pathology

**Figure 2.**
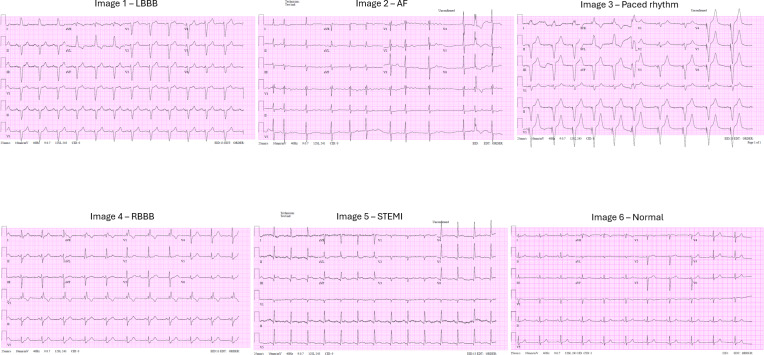
Composite image displaying the 6 electrocardiogram classes used in the multiclass classification few-shot learning approach. AF: atrial fibrillation; LBBB: left bundle branch block; RBBB: right bundle branch block; STEMI: ST-segment elevation myocardial infarction.

This scenario aimed to assess the GPT-4o model’s ability to classify ECG images into specific abnormal categories. The dataset included 60 ECGs, with 10 images from each of 6 pathology classes. Using the zero-shot approach, ECGs were presented without previous examples or guidance (experiments 3.1 and 3.2). In the few-shot learning experiments (experiments 5.1 and 5.2), a single composite image comprising 6 examples (1 from each category) was used, with and without textual guidance. The composite image displaying the 6 pathologies is shown in Figure 2.

### Study End Point

In both the binary (normal or abnormal) and multiclass classification scenarios, GPT-4o’s diagnostic output was compared with the reference assessments made by expert cardiologists who manually reviewed each ECG specifically for this study.

### Evaluation Metrics

The agreement level between the GPT-4o predictions and the actual labels was evaluated using measures of accuracy, sensitivity, specificity, and *F*_1_-score. The positive class was defined as abnormal ECG, with sensitivity representing the detection rate of abnormal ECG, and specificity indicating the detection rate of normal ECG. To ensure the robustness of the results, we repeated the best-performing experiment 5 times and reported both the average values of all evaluation metrics and their corresponding confidence intervals across runs.

### Software and Statistical Analysis

Python (version 3.10; Python Software Foundation) was used to interface with the GPT-4o API and generate visualizations. Statistical analyses and performance metric calculations were conducted using R (version 4.4.2; R Foundation for Statistical Computing).

### Sensitivity Analysis

To assess the robustness of GPT-4o’s performance, we conducted a sensitivity analysis using 2 additional models: a pretrained Vision Transformer (ViT) and Gemini 2.0 Flash (Google), the latest stable version of the Gemini model.

#### Vision Transformer

We implemented a pretrained ViT (vit_base_patch16_224, pretrained on ImageNet) using the timm library in PyTorch. The model was fine-tuned on 10 manually labeled ECG plots (classified as normal or abnormal). Only the classification head was trained, while the transformer backbone remained frozen. Training was performed over 7 epochs using the Adam optimizer (learning rate=1e-4). We also experimented with data augmentation techniques (random rotation and horizontal flipping), which did not improve performance in this small data setting. Model evaluation was performed on a held-out test set of 50 ECG images.

#### Gemini 2.0 Flash

We evaluated Gemini 2.0 flash (Gemini-2.0-Flash-001) using the official Vertex AI SDK (vertexai.generative_models) in Python. Each ECG image was submitted along with the same prompt used in the GPT-4o experiments (as described in the “Methods” section) except for the few-shot learning experiments, which were adapted to the structured format supported by the model. The model’s textual output was parsed to assign a binary class label (normal or abnormal). We assessed accuracy, sensitivity, specificity, and *F*_1_-score using the ground truth labels of the test set. We ran 1 iteration for each experiment and set the temperature parameter to 0.2 for consistency across runs.

### Ethical Considerations

Ethical approval was obtained from the institutional ethics committee following standard institutional procedures (SMC-D-0522-23).

## Results

### Overview

The cohort consisted of 80 patients, with a median age of 69 (IQR 57.0-78.0) years, of which 53.8% (43) were females, carefully selected to ensure representativeness. Table 2 shows the number of patients in each ECG pathology group, the patients’ age distribution, gender, and key ECG parameters that reflect the clinical and electrophysiological diversity of the cohort.

**Table 2.** Demographic characteristics and electrocardiogram parameters of the cohort patients.

Characteristics	Statistics
Total number of patients	80
Group, n (%)	
AF^a^	10 (12.5)
LBBB^b^	10 (12.5)
Normal	30 (37.5)
Paced	10 (12.5)
RBBB^c^	10 (12.5)
STEMI^d^	10 (12.5)
Age at ECG^e^ (years), median (IQR)	69.0 (57.0-78.0)
Sex (female), n (%)	43 (53.8)
Ventricular rate, median (IQR)	72.0 (66.0-81.2)
QRS duration, median (IQR)	98.0 (84.0-138.0)
R axis, median (IQR)	4.5 (−42.8 to 46.2)
T axis, median (IQR)	44.0 (23.2-79.0)
Num QRS complexes, median (IQR)	12.0 (11.0-13.2)
Pacemaker, n (%)	10 (12.5)

a AF: atrial fibrillation.

b LBBB: left bundle branch block.

c RBBB: right bundle branch block.

d STEMI: ST-segment elevation myocardial infarction.

e ECG: electrocardiogram.

As part of a sensitivity analysis, we compared the performance of GPT-4o with Gemini 2.0 Flash and a pretrained ViT model. Since GPT-4o consistently outperformed the alternative models, we report the full sensitivity analysis results in Multimedia Appendix 2. The following sections present the classification results for each scenario using GPT-4o.

### Scenario 1: ECG Image Identification

This scenario assessed the GPT-4o model’s ability to recognize whether an image depicted an ECG. In both simple experiments (experiments 1.1 and 1.2), the model demonstrated excellent recognition ability, correctly classifying 100% of the images as ECG. These findings are consistent with previous work showing that the earlier model, GPT-4V, achieved 100% accuracy in recognizing medical modalities such as ultrasonography, computed tomography, and radiography [23], further supporting GPT-4o’s reliability in fundamental image recognition tasks. However, we did not evaluate its performance in more complex scenarios, such as distinguishing electroencephalograms from ECGs.

### Scenario 2: Distinguishing ECG Images as Normal or Abnormal

This scenario evaluated the GPT-4o model’s ability to differentiate between normal and abnormal ECGs using both zero-shot and few-shot learning approaches. The zero-shot approach showed moderate to high success in diagnosis, with performance gradually improving with the addition of more auxiliary text: 53% without any text, 57% with minimal text, and 63% with extended text (Table 3). The sensitivity in the zero-shot experiments was very high, while the specificity was low, indicating that the model classified most cases as abnormal, including many that were normal. In the initial experiment, where no textual guidance was provided, the specificity was close to zero. Following this, we added the sentence “Normal ECG: Look for regular P waves, QRS complexes, and T waves with consistent intervals between them. Absence of significant abnormalities.” to the prompt, thereby clarifying the definition of a normal ECG. As a result, specificity improved by 26%.

**Table 3.** Scenario 2 results.

Experiment	Technique	Prompt type	Testing size	Accuracy	Sensitivity	Specificity	*F*_1_-score
2.1	Zero-shot	No textual guidance.	60	0.53	1.0	0.07	0.68
2.2	Zero-shot	Minimal textual guidance.	60	0.57	1.0	0.13	0.7
2.3	Zero-shot	Provide textual guidance.	60	0.63	0.93	0.33	0.72
4.2	Few-shot	Learn 6 examples. No textual guidance.	54	0.72	0.67	0.78	0.71
4.3	Few-shot	Learn 6 examples along with added textual guidance.	54	0.8	0.67	0.93	0.77
4.4	Few-shot	Learn 10 examples along with added textual guidance—average results across 5 runs.	50	0.83	0.7	0.97	0.81

**Figure 3.**
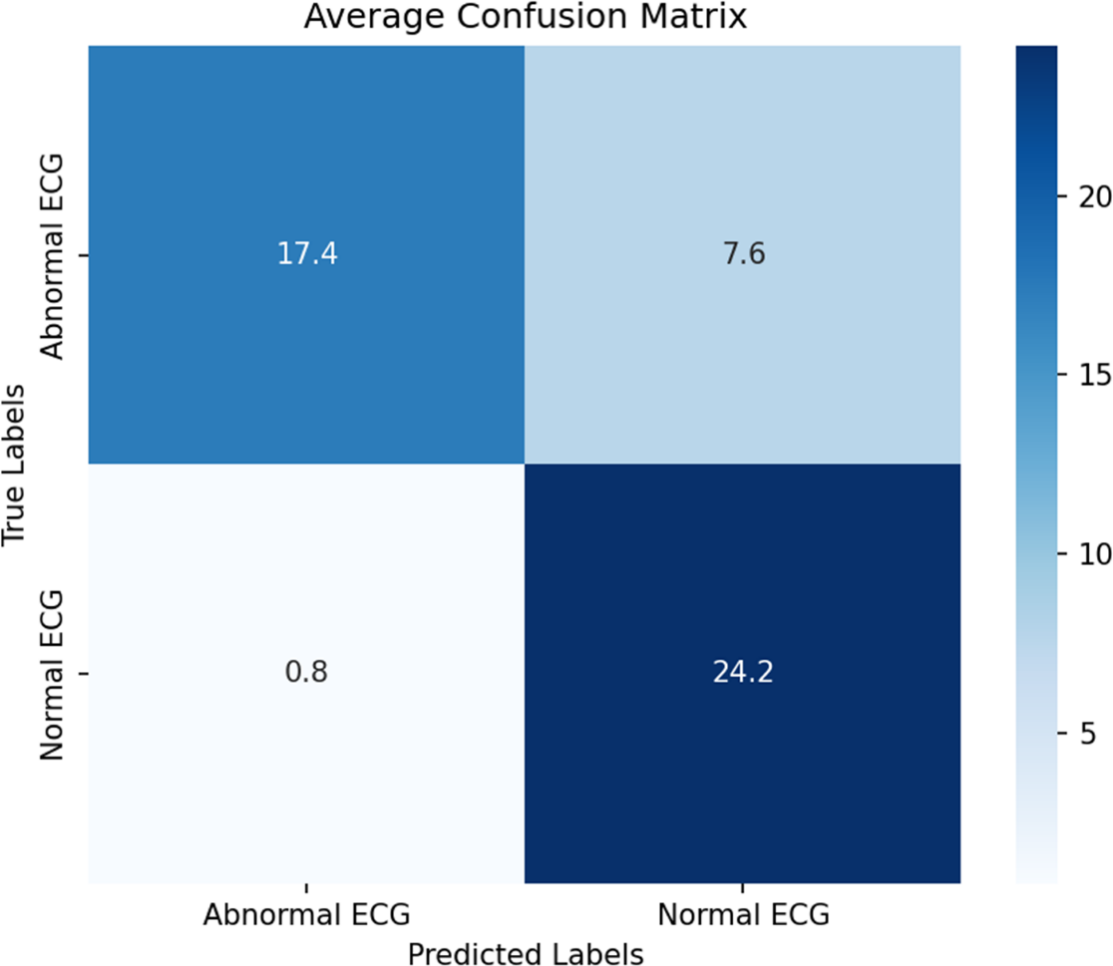
Experiment 4.4 average confusion matrix across 5 iterations. ECG: electrocardiogram.

In contrast, the few-shot approach demonstrated enhanced accuracy, particularly in experiment 4.4. Incorporating 10 learning examples and additional guidance led to the highest classification performance, achieving an average accuracy of 83% (95% CI 81.8%‐84.6%), sensitivity of 70% (95% CI 62.9%‐76.3%), and specificity of 97% (95% CI 92.6%‐100.0%) across 5 runs (Table 3 and Figure 3). By adding textual guidance and providing examples, we improved the accuracy by 30% compared with the baseline model (experiment 2.1), indicating a significant improvement. Multimedia Appendix 3 shows 2 examples of the GPT-4o model’s reasoning when classifying an image as a normal or abnormal ECG. We see from the reason it provides that it considers the R-R intervals, P waves, QRS complex, QRS duration, and T waves. However, the accuracy of these explanations was not formally evaluated in this study.

### Scenario 3: Multiclass Classification for a Specific Pathology

**Figure 4.**
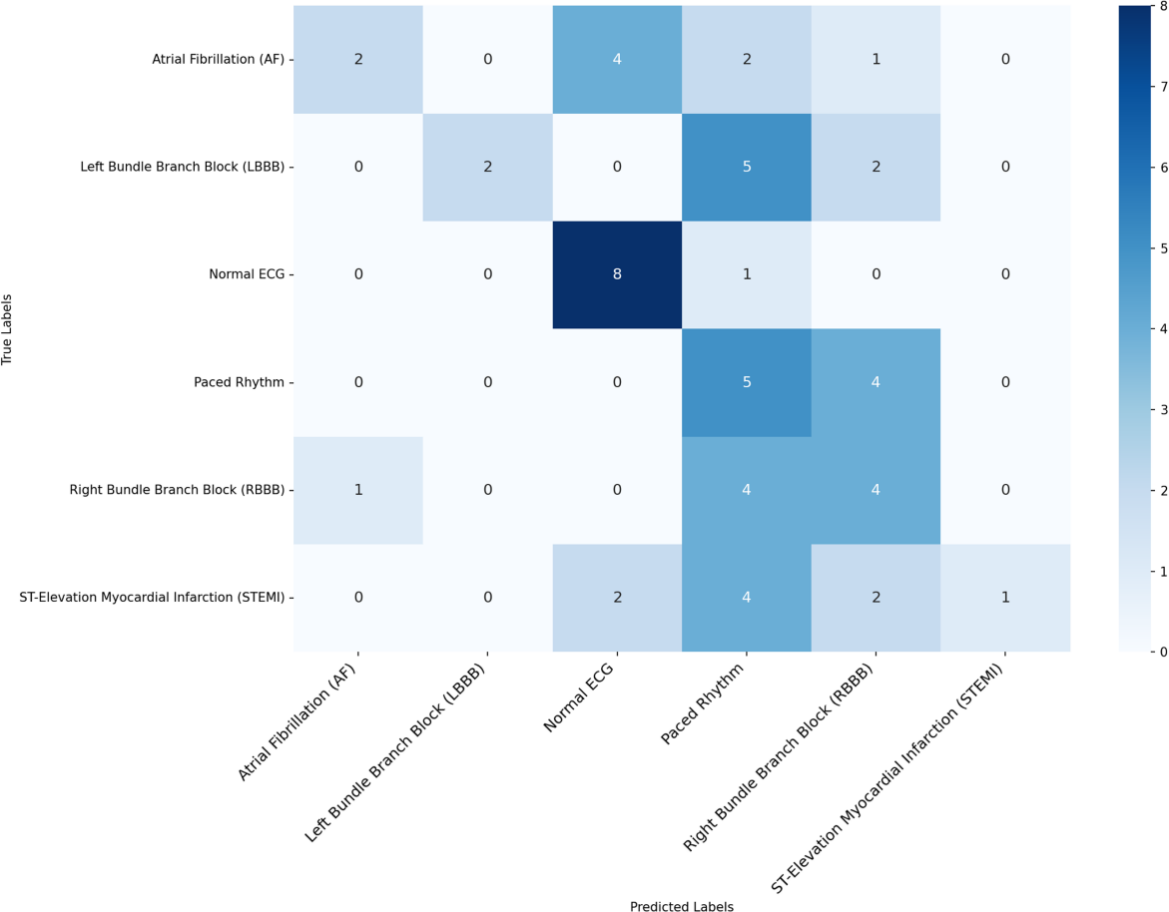
Experiment 5.2 confusion matrix. Multiclass classification, few-shot learning. ECG: electrocardiogram.

In identifying a specific pathology, both approaches showed low success. However, few-shot outperformed zero-shot, achieving an accuracy of 41% compared with 28%. In the few-shot scenario, textual guidance also led to improved results compared with the case without it (Table 4 and Figure 4). Notably, 89% of normal ECGs were correctly classified as normal. Paced rhythm was the most accurately identified cardiac condition, with an accuracy of 55.5%.

**Table 4.** Scenario 3 results.

Experiment	Technique	Prompt type	Testing size	Accuracy
3.1	Zero-shot	No textual guidance.	60	0.28
3.2	Zero-shot	Textual guidance was provided.	60	0.28
5.1	Few-shot	Six examples were provided.	54	0.31
5.2	Few-shot	Six examples were provided along with added textual guidance.	54	0.41

## Discussion

### Principal Findings

This study assesses the image analysis capabilities of GPT-4o for interpreting ECG tests. The main findings reveal that GPT-4o’s capabilities in recognizing and understanding ECG images can be significantly improved with prompt engineering and learning examples. In our case, accuracy improved by 30%. GPT-4o effectively identified the images as ECGs and demonstrated a solid theoretical understanding of ECG components and pathologies. Its performance in distinguishing normal from abnormal ECGs was moderate to high, with an average accuracy of 83% (95% CI 81.8%‐84.6%) across 5 repeated runs on the same 50 ECG examples, reflecting consistent performance. However, the model struggled with more granular classification tasks, achieving only 41% accuracy when identifying specific diagnoses. Furthermore, the study showed that few-shot learning surpassed zero-shot learning, and combining textual instructions with image examples led to better outcomes, achieving moderate to high accuracy and high specificity improvement compared with the baseline model. As part of a sensitivity analysis to contextualize GPT-4o’s performance, we also evaluated Gemini 2.0 Flash and a pretrained ViT model; however, neither outperformed GPT-4o in this task.

Previous studies [24-31] extensively investigated DL AI models’ diagnostic capabilities for classifying ECGs, achieving higher accuracy rates compared with our study, which explored the performance of LLMs in zero-shot and few-shot learning contexts. While previous studies have reported superior accuracy using specialized DL models (eg, CNNs and LCNNs), these approaches require substantial computational resources and model-specific training, limiting their accessibility in routine clinical practice. In contrast, multimodal LLMs such as GPT-4o provide a low-barrier alternative that could support medical professionals without specialized AI expertise.

Our findings also align with recent research on the robustness of multimodal models to domain shifts, such as ECG images, which differ substantially from the natural images seen during model pretraining. Previous work has shown that performance under such shifts can be improved through in-context learning strategies such as few-shot learning, as demonstrated in studies evaluating GPT-4V and other vision-language models [32-35]. In our study, this was evident in the improved performance observed with few-shot learning when distinguishing normal from abnormal ECGs. However, the model continued to struggle with identifying specific pathologies, as seen in scenario 3. Several factors likely contributed to this limitation. Certain cardiac conditions are inherently difficult to detect, as their features may be masked by noise, artifacts, or subtle waveform variations [16]. These factors can mislead the model, especially with incomplete or atypical ECGs that do not match the patterns it learned during training [36], situations in which multimodal LMMs often fail to generalize effectively. Furthermore, the absence of clinical context may further constrain performance, as incorporating patient symptoms or medical history has been shown to enhance diagnostic accuracy [37]. Together, these factors likely contributed to the model’s limited ability to accurately identify specific abnormalities.

When comparing our study with those investigating AI’s diagnostic performance, a distinct contrast emerges. These studies, using DL models trained on large ECG datasets for specific diagnosis tasks ranging from arrhythmia to STEMI detection, consistently report high diagnostic accuracy rates, often exceeding 90% [24-29]. Conversely, compared with the studies focusing on binary classification of ECGs (normal vs abnormal) [30,31], our study achieved a moderate to high accuracy of 83% despite minimal training, and by that, highlighting the potential of accessible AI models for cardiac diagnostics. Conversely, compared with the studies focusing on binary classification of ECGs (normal vs abnormal) [30,31], our study achieved a moderate to high accuracy of 83% despite minimal training, and by that, highlighting the potential of accessible AI models for cardiac diagnostics.

In addition to its potential in cardiology, GPT-4o’s image interpretation capabilities find relevance in various medical domains, such as radiology, neurology, and ophthalmology. Research in these fields indicates that while GPT-4o can identify imaging modalities and tackle intricate diagnostic tasks, its current success rates remain modest [14-17,33].

Consistent with these findings, our results suggest that although GPT-4o shows promise in medical image interpretation, it remains best suited as a supplementary tool to support, rather than replace, clinical expertise [15,16]. This is especially important given the risk of hallucinations and overconfident misclassifications that LLMs may produce when faced with ambiguous or unfamiliar inputs [38]. As multimodal AI models continue to evolve, further research is needed to refine their integration into diagnostic workflows and optimize their clinical use.

### Limitations and Future Research

The current findings rely on a small retrospective sample of 80 patients. While this limited sample size constrains the statistical robustness of the findings, it was sufficient to support a focused proof-of-concept evaluation of GPT-4o’s capabilities in ECG interpretation. The sample, although small, demonstrated consistent performance across repeated runs and helped highlight key challenges and opportunities in applying multimodal LLMs to ECG analysis. Moreover, our study acknowledges the documented potential impact of prompt wording variations on GPT-4o’s responses [39]. Minor changes in prompts can significantly affect language models such as GPT-4o. Finally, our study solely evaluated GPT-4o with ECG recordings, excluding the patient’s medical history, a departure from typical clinical practice, where attending physicians have access to comprehensive patient information. We hypothesize that incorporating these contextual data into the model could enhance diagnostic accuracy.

Future research could assess custom GPT-4o performance when it is enhanced with specific knowledge sources, such as cardiology textbooks, rather than solely instructions. Furthermore, to address the challenges observed in multiclass classification of specific diagnoses (scenario 3, Multimedia Appendix 4), future studies should explore few-shot learning setups that include multiple examples for each diagnostic class and test on a larger sample. As demonstrated in previous work, this approach can improve performance under domain shift conditions by enabling the model to generalize more effectively across diverse pathology patterns [36,40]. Finally, future work should consider evaluating more advanced models such as Gemini 2.5, which, while not yet part of a stable public release, has demonstrated strong performance in multimodal tasks and may offer improved capabilities for clinical ECG interpretation.

### Conclusions

The current version of GPT-4o exhibits moderate to high proficiency in distinguishing between normal and abnormal ECG readings. However, its ability to diagnose specific cardiac conditions remains limited. Our findings suggest that GPT-4o’s performance can be enhanced through prompt engineering and few-shot learning, highlighting its potential as a supplementary decision support system in clinical practice. Future improvements to the algorithm, particularly in fine-tuning its diagnostic capabilities, could further expand its use in medical image analysis.

## Supplementary material

10.2196/74426 Multimedia Appendix 1 The prompt of each experiment.

10.2196/74426 Multimedia Appendix 2 Sensitivity analysis.

10.2196/74426 Multimedia Appendix 3 Examples of the GPT-4o reasoning when deciding whether an electrocardiogram is normal or abnormal.

10.2196/74426 Multimedia Appendix 4 Illustration of the challenges in classifying specific pathologies within a few-shot learning setup.

## Abbreviations

AF: atrial fibrillation
AI: artificial intelligence
CNN: convolutional neural network
DL: deep learning
ECG: electrocardiogram
GPT-4o: Generative Pre-trained Transformer 4 Omni
LBBB: left bundle branch block
LLM: large language model
RBBB: right bundle branch block
STEMI: ST-segment elevation myocardial infarction
ViT: Vision Transformer
