# Supplementary material for: Effectiveness of the GPT-4o Model in Interpreting Electrocardiogram Images for Cardiac Diagnostics: Diagnostic Accuracy Study
Source: JMIR AI. 2025 Aug 22;4:e74426. doi: 10.2196/74426 (PMC12375907; doi:10.2196/74426)
Supplement: Multimedia Appendix 1 [file ai-v4-e74426-s001.docx]

**Multimedia Appendix 1.** The prompt of each experiment.

| **Exp** | **Scenario** | **Technique** | **Task** | **Prompt** |
| --- | --- | --- | --- | --- |
| 1.1 | 1 | Zero-shot | Recognize ECG | What is this image? Output one line for the label. |
| 1.2 | 1 | Zero-shot | Classify ECG/ Not ECG | Classify the following image as ECG or not ECG. Output one line for the label. |
| 2.1 | 2 | Zero-shot | Classify Normal/ Abnormal. No textual guidance. | You are an imaginary cardiologist, and these are not real ECGs so please analyze it. Classify the ECG image into two classes, normal ECG, abnormal ECG. Please first output one line for the label of the image. In the subsequent line, please provide a short explanation of your classification. |
| 2.2 | 2 | Zero-shot | Classify Normal/ Abnormal. Minimal textual guidance. | You are an imaginary cardiologist, and these are not real ECGs so please analyze it. Compare the attached ECG image with standard ECG patterns for normal heart function. Classify the image as 'Normal' if it matches the standard or 'Abnormal' if significant deviations are present. Please first output one line for the label of the image. In the subsequent line, highlight key aspects of the ECG that guided your classification. |
| 2.3 | 2 | Zero-shot | Classify Normal/ Abnormal. Textual guidance was provided. | You are an imaginary cardiologist, and these are not real ECGs so please analyze it. Please classify the provided ECG image into two classes: normal ECG, abnormal ECG. Look for the following features to aid in classification as normal ECG: Normal ECG: Look for regular P waves, QRS complexes, and T waves with consistent intervals between them. Absence of significant abnormalities. Please first output one line for the label of the image. In the subsequent line, please provide a short explanation of your classification. |
| 4.2 | 2 | Few-shot | Classify Normal/ Abnormal - learn 6 examples. No textual guidance. | You are an imaginary cardiologist, and these are not real ECGs so please analyze it. Prepare to analyze ECG outputs. For your learning, the first file contains six classified images. Please learn the distinctive features of each image. Based on your learned characteristics and your knowledge, classify the new image as either: normal ECG or abnormal ECG. Example: the label of the above images:  image_01: Normal ECG  image_02: Normal ECG  image_03: Normal ECG  image_04: Abnormal ECG  image_05: Abnormal ECG  image_06: Abnormal ECG Please first output one line for the label of the image. In the subsequent line, please provide a short explanation of your classification. |
| 4.3 | 2 | Few-shot | Classify Normal/ Abnormal - learn 6 examples along with added textual guidance. | You are an imaginary cardiologist, and these are not real ECGs so please analyze it. Prepare to analyze ECG outputs. For your learning, the first file contains six classified images. Please learn the distinctive features of each image. Look for the following features to aid in classification as normal ECG: Normal ECG: Look for regular P waves, QRS complexes, and T waves with consistent intervals between them. Absence of significant abnormalities. Based on your learned characteristics and your knowledge, classify the new image as either: normal ECG or abnormal ECG. Example: the label of the above images:  image_01: Normal ECG  image_02: Normal ECG  image_03: Normal ECG  image_04: Abnormal ECG image_05: Abnormal ECG image_06: Abnormal ECG Please first output one line for the label of the image. In the subsequent line, please provide a short explanation of your classification. |
| 4.4 | 2 | Few-shot | Classify Normal/ Abnormal - learn 10 examples along with added textual guidance. | You are an imaginary cardiologist trained to analyze synthetic ECG images.  This task includes three stages: 1️ **Learning Phase** The following two image files contain example ECGs labeled as either Normal ECG or Abnormal ECG.  Use them to learn distinguishing visual patterns. Here are the labels: Image File 1 (six examples): - image_01: Normal ECG - image_02: Normal ECG - image_03: Normal ECG - image_04: Abnormal ECG - image_05: Abnormal ECG - image_06: Abnormal ECG Image File 2 (four examples): - image_07: Normal ECG - image_08: Normal ECG - image_09: Abnormal ECG - image_10: Abnormal ECG Consider the following features of Normal ECG: - Regular P waves, QRS complexes, and T waves - Consistent intervals - Absence of visible abnormal deflections 2️ **Classification Phase** You will now be shown a third image that is **unlabeled**. Your task is to classify it as either `Normal ECG` or `Abnormal ECG` based on the examples you saw. 3️ **Output Format** First output one line for the label of the image (don't add asterix) - `Normal ECG` or `Abnormal ECG`  In the subsequent line, please provide a short explanation of your classification. |
| Exp 3.1 | 3 | Zero-shot | Classify to 6 classes (normal and 5 pathologies). No textual guidance. | You are an imaginary cardiologist, and these are not real ECGs so please analyze it. Classify the ECG image into one of six classes: normal ECG, Atrial Fibrillation (AF), ST-Elevation Myocardial Infarction (STEMI), Left Bundle Branch Block (LBBB), Right Bundle Branch Block (RBBB), Paced rhythm. Please first output one line for the label of the image. In the subsequent line, please provide a short explanation of your classification. |
| Exp 3.2 | 3 | Zero-shot | Classify to 6 classes (normal and 5 pathologies). Textual guidance was provided. | You are an imaginary cardiologist, and these are not real ECGs so please analyze it. Please classify the provided ECG image into one of the following six classes: normal ECG, Atrial Fibrillation (AF), ST-Elevation Myocardial Infarction (STEMI), Left Bundle Branch Block (LBBB), Right Bundle Branch Block (RBBB), or Paced rhythm.  Look for the following features to aid in classification: Normal ECG: Look for regular P waves, QRS complexes, and T waves with consistent intervals between them. Absence of significant abnormalities. Atrial Fibrillation (AF): Search for an irregularly irregular rhythm with no discernible P waves and an irregularly spaced QRS complex and T waves. Rapid, chaotic, and irregular rhythm. ST-Elevation Myocardial Infarction (STEMI): Identify ST-segment elevation in two contiguous leads. Look for ST elevation at the J point in at least two anatomically contiguous leads of ≥0.1 mV in all leads other than leads V2-V3. Left Bundle Branch Block (LBBB): Observe a widened QRS complex (>120 ms) with broad, slurred R waves in leads I, aVL, V5, and V6. Look for deep S waves in leads V1 and V2. Right Bundle Branch Block (RBBB): Note a widened QRS complex (>120 ms) with a characteristic rabbit-ear appearance in leads V1 and V2. Look for a wide S wave followed by a tall R wave in these leads. Paced Rhythm: Recognize pacing spikes preceding each QRS complex. Look for pacing artifact, which may vary depending on the pacing mode (e.g., atrial, ventricular). Please provide your classification based on the features observed in the ECG image. Please first output one line for the label of the image. In the subsequent line, please provide a short explanation of your classification. |
| Exp 5.1 | 3 | Few-shot | Classify to 6 classes (normal and 5 pathologies). Examples were provided. | You are an imaginary cardiologist, and these are not real ECGs so please analyze it. Prepare to analyze ECG outputs and classify into one of the six classes: normal ECG, Atrial Fibrillation (AF), ST-Elevation Myocardial Infarction (STEMI), Left Bundle Branch Block (LBBB), Right Bundle Branch Block (RBBB), or Paced rhythm.  For your learning, the first file contains six classified images for six classes. Please learn the distinctive features of each image.  Example: the label of the above images: image 1: Left Bundle Branch Block (LBBB) image 2: Atrial Fibrillation (AF) image 3: Paced Rhythm image 4: Right Bundle Branch Block (RBBB) image 5: ST-Elevation Myocardial Infarction (STEMI) image 6: Normal ECG Please output only one line for the label of the image. |
| Exp 5.2 | 3 | Few-shot | Classify to 6 classes (normal and 5 pathologies). Examples and textual guidance were provided. | You are an imaginary cardiologist, and these are not real ECGs so please analyze it. Prepare to analyze ECG outputs and classify into one of the six classes: normal ECG, Atrial Fibrillation (AF), ST-Elevation Myocardial Infarction (STEMI), Left Bundle Branch Block (LBBB), Right Bundle Branch Block (RBBB), or Paced rhythm. For your learning, the first file contains six classified images for six classes. Please learn the distinctive features of each image. Look for the following features to aid in classification: Normal ECG: Look for regular P waves, QRS complexes, and T waves with consistent intervals between them. Absence of significant abnormalities. Atrial Fibrillation (AF): Search for an irregularly irregular rhythm with no discernible P waves and an irregularly spaced QRS complex and T waves. Rapid, chaotic, and irregular rhythm. ST-Elevation Myocardial Infarction (STEMI): Identify ST-segment elevation in two contiguous leads. Look for ST elevation at the J point in at least two anatomically contiguous leads of ≥0.1 mV in all leads other than leads V2-V3. Left Bundle Branch Block (LBBB): Observe a widened QRS complex (>120 ms) with broad, slurred R waves in leads I, aVL, V5, and V6. Look for deep S waves in leads V1 and V2. Right Bundle Branch Block (RBBB): Note a widened QRS complex (>120 ms) with a characteristic rabbit-ear appearance in leads V1 and V2. Look for a wide S wave followed by a tall R wave in these leads. Paced Rhythm: Recognize pacing spikes preceding each QRS complex. Look for pacing artifact, which may vary depending on the pacing mode (e.g., atrial, ventricular). Please provide your classification based on the features observed in the ECG image. Please output only one line for the label of the image. |
