# Supplementary material for: Effectiveness of the GPT-4o Model in Interpreting Electrocardiogram Images for Cardiac Diagnostics: Diagnostic Accuracy Study
Source: JMIR AI. 2025 Aug 22;4:e74426. doi: 10.2196/74426 (PMC12375907; doi:10.2196/74426)
Supplement: Multimedia Appendix 2 [file ai-v4-e74426-s002.docx]

**Multimedia Appendix 2. Sensitivity Analysis.**

**1. Pretrained Vision Transformer (ViT) performance**

| **Model** | **Testing size** | **Accuracy** | **Sensitivity** | **Specificity** | **F1** |
| --- | --- | --- | --- | --- | --- |
| vit_base_patch16_224 | 50 | 0.78 | 0.84 | 0.72 | 0.79 |

**2. Gemini 2.0 flash**

**Gemini 2.0 flash performance for the second scenario – classifying ECG as normal or abnormal**

| **Exp** | **Technique** | **Prompt type** | **Testing size** | **Accuracy** | **Sensitivity** | **Specificity** | **F1** |
| --- | --- | --- | --- | --- | --- | --- | --- |
| 2.1 | Zero-shot | No textual guidance. | 60 | 0.53 | 0.97 | 0.1 | 0.67 |
| 2.2 | Zero-shot | Minimal textual guidance. | 60 | 0.53 | 1.0 | 0.07 | 0.68 |
| 2.3 | Zero-shot | Provide textual guidance. | 60 | 0.63 | 0.77 | 0.5 | 0.68 |
| 4.2 | Few-shot | Learn 6 examples. No textual guidance. | 54 | 0.54 | 0.93 | 0.15 | 0.67 |
| 4.3 | Few-shot | learn 6 examples along with added textual guidance. | 54 | 0.59 | 0.85 | 0.33 | 0.68 |
| 4.3 | Few-shot | The prompt structure was adapted to Gemini few-shot formatting. Learn 6 examples along with added textual guidance. | 54 | 0.7 | 0.85 | 0.56 | 0.74 |
| 4.4 | Few-shot | The prompt structure was adapted to Gemini few-shot formatting. Learn 10 examples along with added textual guidance. | 50 | 0.8 | 0.85 | 0.74 | 0.8 |

**Gemini 2.0 flash performance for the third scenario – Multiclass classification for specific pathologies**

Using Gemini 2.0 Flash, the few-shot approach outperformed the zero-shot setting, achieving an accuracy of 35% versus 27%. While the overall accuracies in scenarios 5.1 and 5.2 were similar, class-wise performance differed slightly, with scenario 5.2 showing stronger classification for the classes it identified more accurately. Notably, 89% of normal ECGs were correctly classified as normal. Atrial fibrillation was the most accurately detected condition, reaching 67% accuracy in this scenario.

| **Exp** | **Technique** | **Prompt type** | **Testing size** | **Accuracy** |
| --- | --- | --- | --- | --- |
| 3.1 | Zero-shot | No textual guidance | 60 | 0.27 |
| 3.2 | Zero-shot | Textual guidance was provided. | 60 | 0.23 |
| 5.1 | Few-shot | Six examples were provided. | 54 | 0.35 |
| 5.2 | Few-shot | Six examples were provided along with added textual guidance | 54 | 0.35 |
