# Supplementary material for: Effectiveness of the GPT-4o Model in Interpreting Electrocardiogram Images for Cardiac Diagnostics: Diagnostic Accuracy Study
Source: JMIR AI. 2025 Aug 22;4:e74426. doi: 10.2196/74426 (PMC12375907; doi:10.2196/74426)
Supplement: Multimedia Appendix 3 [file ai-v4-e74426-s003.docx]

**Multimedia Appendix 3. Examples of the GPT-4o reasoning when deciding whether an ECG is normal or abnormal.**

**[B1] Image #107: Normal ECG**


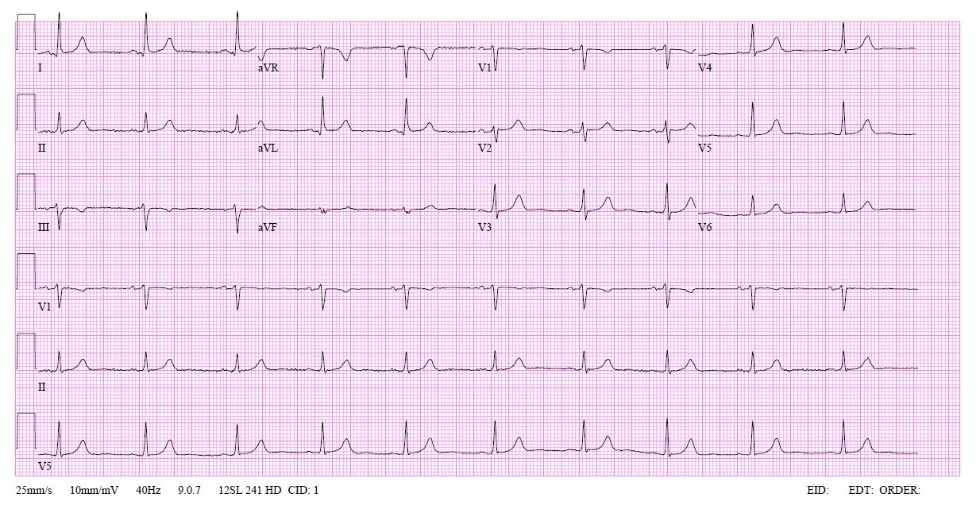


*The ECG shows regular P waves, QRS complexes, and T waves with consistent intervals between them, indicating a normal rhythm without significant abnormalities.*

**[B2] Image #18:** **Abnormal ECG**


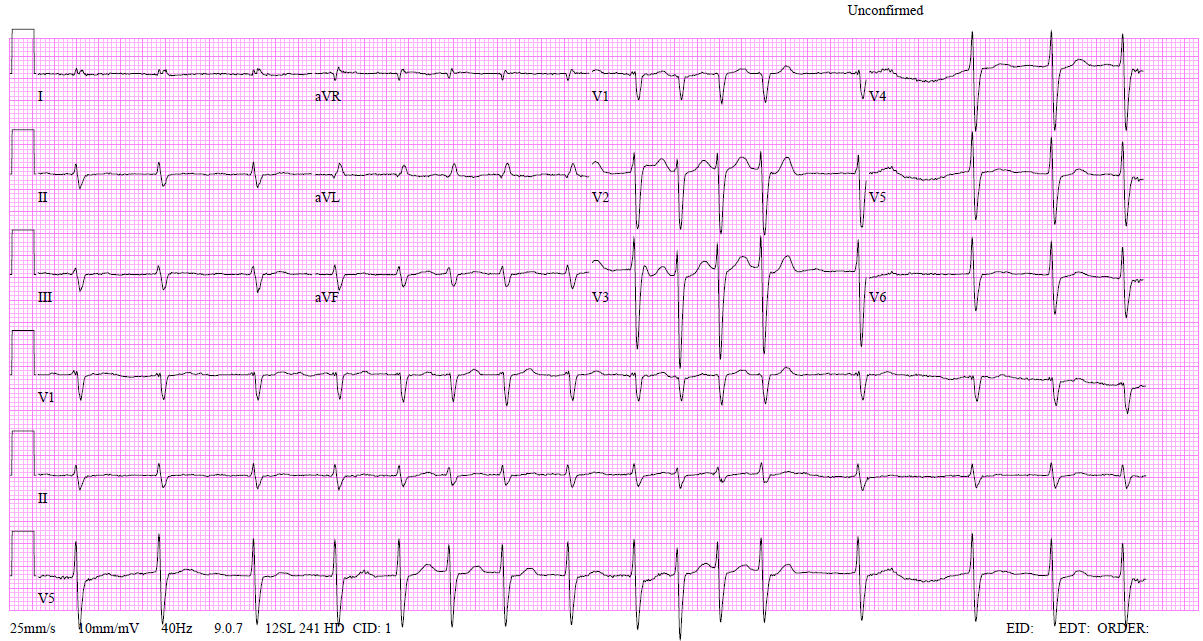


*The ECG shows irregularities such as abnormal wave patterns, inconsistent intervals, or unusual shapes in the QRS complexes, indicating a deviation from a normal ECG.*
