# Supplementary material for: Effectiveness of the GPT-4o Model in Interpreting Electrocardiogram Images for Cardiac Diagnostics: Diagnostic Accuracy Study
Source: JMIR AI. 2025 Aug 22;4:e74426. doi: 10.2196/74426 (PMC12375907; doi:10.2196/74426)

**Multimedia Appendix 4. Illustration of the challenges in classifying specific pathologies within a few-shot learning setup.**

This appendix illustrates the challenges GPT-4o faced in classifying specific pathologies using only a single learning example per class in a few-shot learning setup. The examples below show how intra-class morphological variability can lead to misclassification, even when typical diagnostic features are present.

- Image D1 presents the provided learning example for left bundle branch block (LBBB), characterized by prolonged QRS duration, deep S waves in leads V1-V3, and broad, notched R waves in lateral leads (V5-V6).
- Images D2-D3 are LBBB cases that were misclassified as paced rhythm. Right ventricular pacing, usually originating from a septo-apical lead position, does result in an LBBB-like morphology in V1 and broad QRS complexes, making the distinction from true LBBB challenging. The fact that visible pacing spikes appear reliably only in unipolar pacing, an uncommon feature in contemporary pacing systems, further increases the difficulty of distinguishing between the two. Yet important morphological differences do exist. Chief among them are an abnormal axis (superior or leftward-directed), a late transition on the precordial leads, and often notches in the lateral leads. Both ECGs are defined by a normal axis and do display notches more typical of LBBB. This highlights the model’s reliance on limited shape-based heuristics rather than a more comprehensive view of the entire tracing, resulting in its difficulty distinguishing LBBB from paced rhythms in the absence of pacing artifacts.
- Image D4 is an LBBB ECG that was misclassified as right bundle branch block (RBBB). While it clearly demonstrates classic LBBB morphology, the model may have been misled by the ST depression and T wave inversion in lead I, misclassifying it as deep S waves typical of RBBB. It may have also been misled by the early R wave deflections in lead V1. This may be the result of insufficient variation in the LBBB learning examples. Unlike RBBB, there is no terminal R’ in V1 and no rsR’ pattern.

These misclassifications underscore the importance of providing multiple, morphologically diverse examples per class in few-shot learning setups. They also suggest that general-purpose vision-language models like GPT-4o may struggle with subtle distinctions between ECG subtypes, especially when visual differences are nuanced or overlap with other rhythm morphologies.

**[D1] Image #1 - LBBB learning example**

**
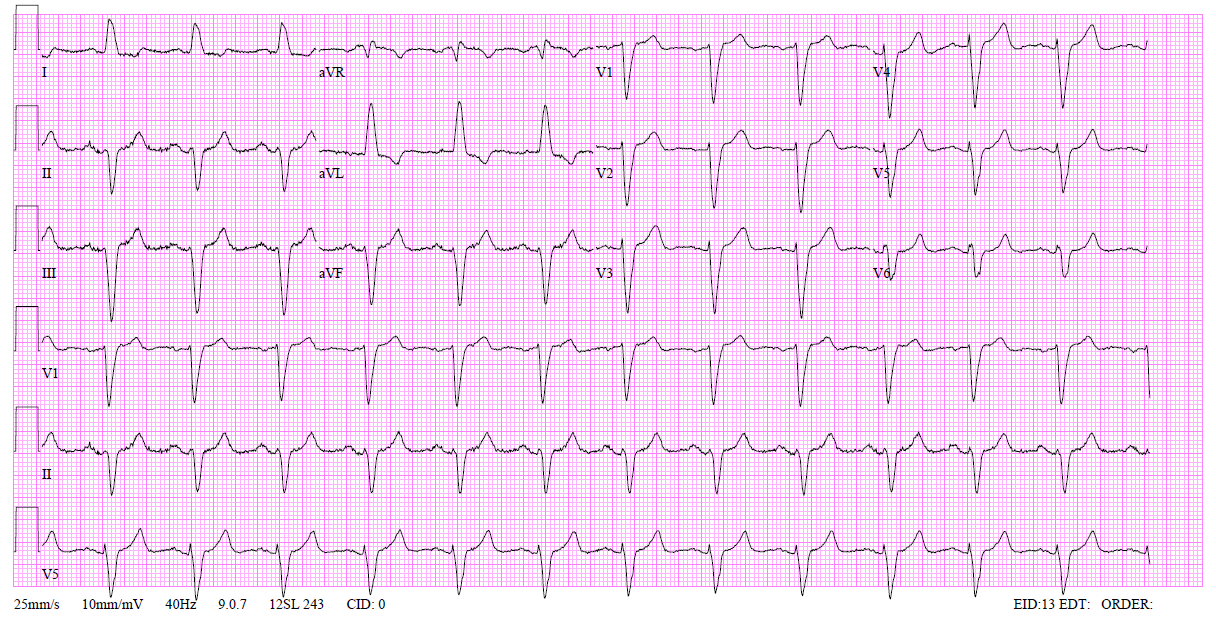
**

**LBBB images misclassified by the model**

**[D2] Image #3 – image label is LBBB, was classified to Paced Rhythm**


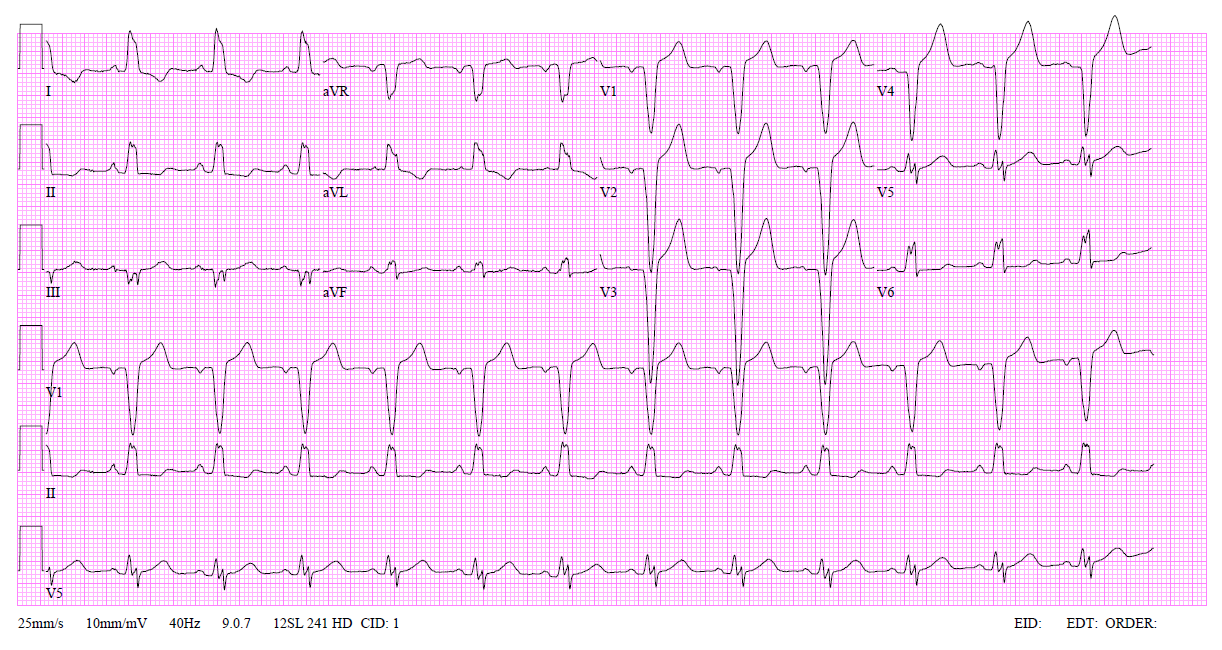


**[D3] Image #4 – image label is LBBB, was classified to Paced Rhythm**


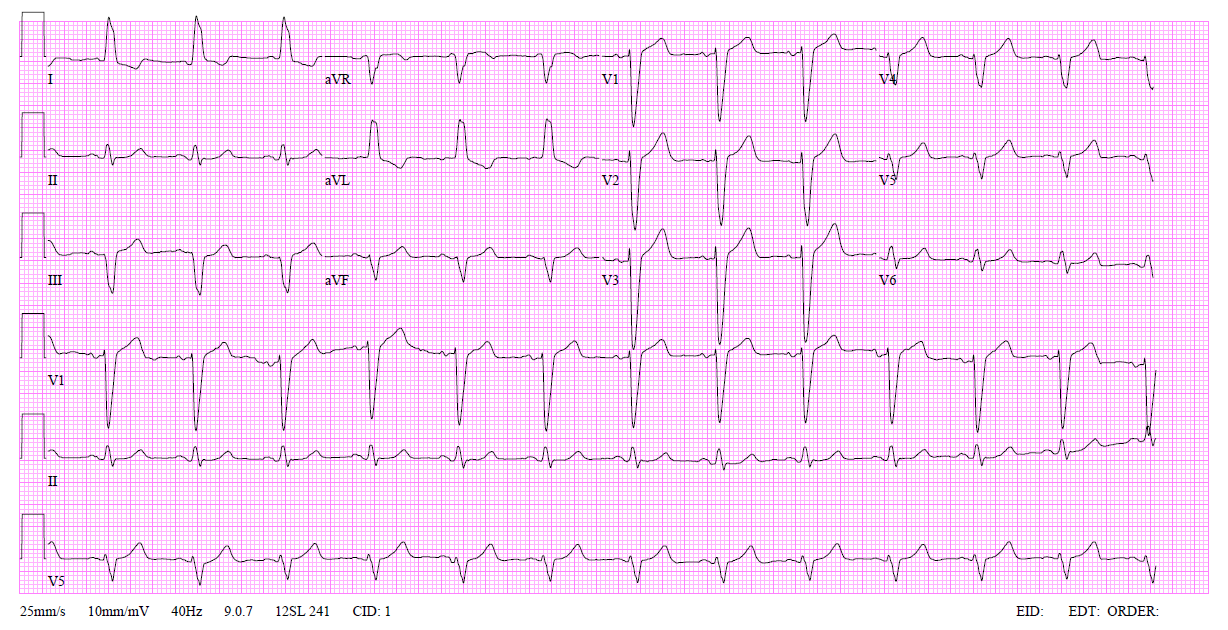


**[D4] Image #5 – image label is LBBB, was classified to RBBB**


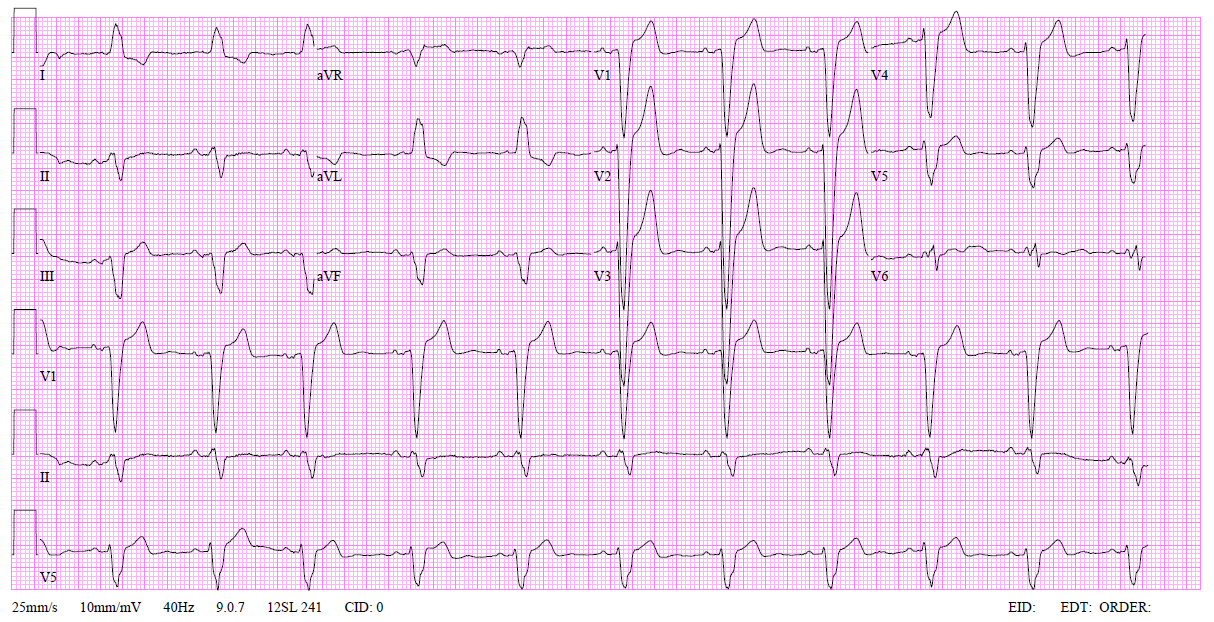

Supplement: Multimedia Appendix 4 [file ai-v4-e74426-s004.docx]
